# Comprehensive Analysis of the Expression Profiles of Long Non-Coding RNAs with Associated ceRNA Network Involved in the Colon Cancer Staging and Progression

**DOI:** 10.1038/s41598-019-52883-2

**Published:** 2019-11-15

**Authors:** Meini Wu, Wenliang Li, Fengchang Huang, Jing Sun, Kang ping Li, Jiandong Shi, Jingyu Yang, Jianfang Li, Yanhan Li, Ningzhu Hu, YunZhang Hu

**Affiliations:** 1 Department of vaccinology, Institute of Medical Biology, Chinese Academy of Medical Sciences and Peking Union Medical College, Kunming, 650118 China; 2 Department of Surgical Oncology, First Affiliated Hospital of Kunming Medical University, Kunming, China

**Keywords:** Non-coding RNAs, Cancer genomics

## Abstract

Long non-coding RNAs (lncRNAs) act as competing endogenous RNAs (ceRNAs) to compete with microRNAs (miRNAs) in cancer occurrence and development. However, the differential expression of RNAs and their ceRNA network during the development of colon cancer (CC) remains unclear. This study was aimed at comprehensive analysis of the lncRNAs and their ceRNA networks associated with CC. Whole transcriptome sequencing was performed on colorectal and adjacent normal tissues at different pathological stages. Forty-nine lncRNAs were differently expressed between the CC tissues and their adjacent normal tissues at all stages. Aberrant expression of lncRNA CDKN2B-AS1 and lncRNA MIR4435-2HG was confirmed by TCGA database. Moreover, 14 lncRNAs were differentially expressed between early and advance stages of the tumor tissues, and 117 miRNAs were specifically expressed in stage III & IV. Weighted gene co-expression network analysis of 17105 differently expressed mRNAs revealed that the mRNAs shown in module pink, midnight blue, black, and light cyan were related to TNM and pathological stage, and that these mRNAs were enriched in cancer related functions and pathways. As DElncRNA showed a trend of change similar to that of the DEmRNA and opposite to that of DEmiRNA, ceRNA network was constructed with 3 DEmiRNAs, 5 DElncRNAs, and 130 DEmRNAs. Real time PCR revealed that expression of MEG3 was decreased in the tumor tissues belonging to stage III and IV as compared to that in stage I. Moreover, hsa-miR-324-5p was upregulated, while FGFR3, PLCB4, and IKBKB were downregulated in the tumor tissues as compared to that in the adjacent normal tissues. Thus, this study revealed differentially expressed lncRNA between different stages of CC as well as suggested that lncRNA CDKN2B-AS1, MIR4435-2HG, and MEG3 may act as diagnostic biomarkers for the development of CC.

## Introduction

Colon cancer (CC)^1^ is one of the most prevalent cancers worldwide; it is also the second leading cause of mortality associated with cancer^2,3^. CC carcinogenesis, a multi-step process, occurs with the accumulation of various genetic or epigenetic alternations^4^. Currently, the most routinely utilized maker in clinical practice for metastatic CC patients is the tumor mutated KRAS gene, which predicts non-response to anti-EGFR antibodies such as cetuximab^5^. Survival rates of patients with CC have risen up in the past few years, which might be a consequence of earlier diagnosis and prognosis. In this study, we aimed to provide a more systematic analysis of factors or markers for the diagnosis and treatment of CC.

Thousands of non-coding transcripts, including non-coding linear RNAs microRNAs(miRNAs), long non-coding RNAs (lncRNA), and circular RNAs (circRNA), involved in various diseases as well as cancers have been identified with the advances in the RNA-sequencing techniques^6^. The lncRNAs are functionally defined as transcripts, which are more than 200 nucleotides in length with no protein coding potential^7^; most of these are characteristically expressed in specific tissues or cancer types and possess conserved secondary structure and function^8^. A number of lncRNAs could act as diagnosis and prognosis markers, and have potential of being therapeutic targets for tumors. A recent study found that lncRNA TTTY15 was frequently up-regulated in a large group of prostate cancer (PCa) tissues. The TTTY15 knockout showed a tumor suppressive effect, which might have therapeutic implications for PCa patients^9^. Most studies focus on the mechanism of the action of lncRNA as competing RNA for miRNA as well as its competitive binding to the miRNA targets, which is called as ceRNA mechanism^10^. For instance, Long noncoding RNA LINC01234 was identified to function as a competing endogenous RNA and sponging miR-204-5p; the CBFB expression was regulated by gain or loss of expression of LINC01234, thus, affecting the gastric cancer tumorigenesis^11^.

A variety of lncRNAs have also been reported to be involved in CC development. For instance, a recent study revealed that nuclear lncRNA HOXD-AS1 inhibited colorectal cancer proliferation and metastasis by regulating HOXD3-induced integrin β3 transcriptional activity, thus inhibiting MAPK/AKT signaling^12^. When considering the ceRNA mechanism, a study also identified that MALAT1 acted as a sponge of miR-106b-5p to competitively bind to SLAIN2, which enhanced the microtubules mobility to promote the invasion and metastasis of colorectal carcinoma^13^. However, there has been no comprehensive analysis of CC-related lncRNAs and miRNAs based on ceRNA networks combined with large comprehensive analysis of the pathological characteristics of patients with CC.

## Results

### Identification of differentially expressed (DE) lncRNAs

**Figure 1:**
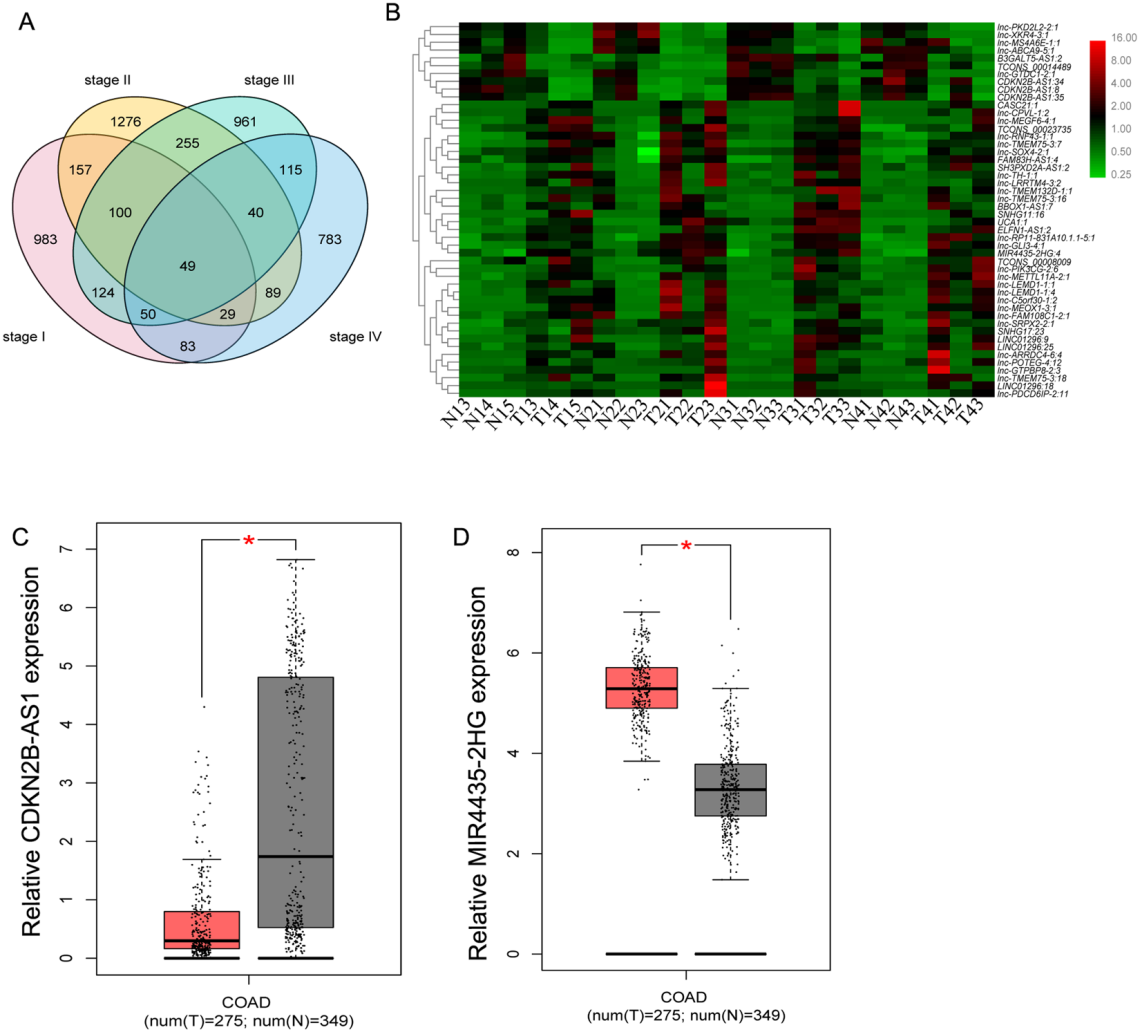
Differentially expressed (DE) lncRNAs between gastric cancer tissues and corresponding adjacent normal tissues. (**A**) Venn diagram analysis of DE lncRNAs at four stages of CC. (**B**) Heatmaps of DElncRNAs in CC. The red and green colors represent up- and down-regulation, respectively. Expression of lncRNA CDKN2B-AS1(**C**) and MIR4435-2HG (**D**) in colon adenocarcinoma (COAD).

A total of 49 DElncRNAs were shared in all stages between the CC tissues and their adjacent normal tissues (n = 3); among these, 48 DElncRNAs shared the same trend of up- or down-regulation in all stages, including 10 down-regulated and 38 up-regulated lncRNAs in the CC tissue. The significant difference was defined as the log2 (fold change) >1 or log2 (fold change) <−1 and p-value < 0.05 (Fig. 1A,B). Among them, the expression of down-regulated lncRNA CDKN2B-AS1 and the up-regulated lncRNA MIR4435-2HG was confirmed by comparing with the TCGA database (Fig. 1C,D).

**Figure 2:**
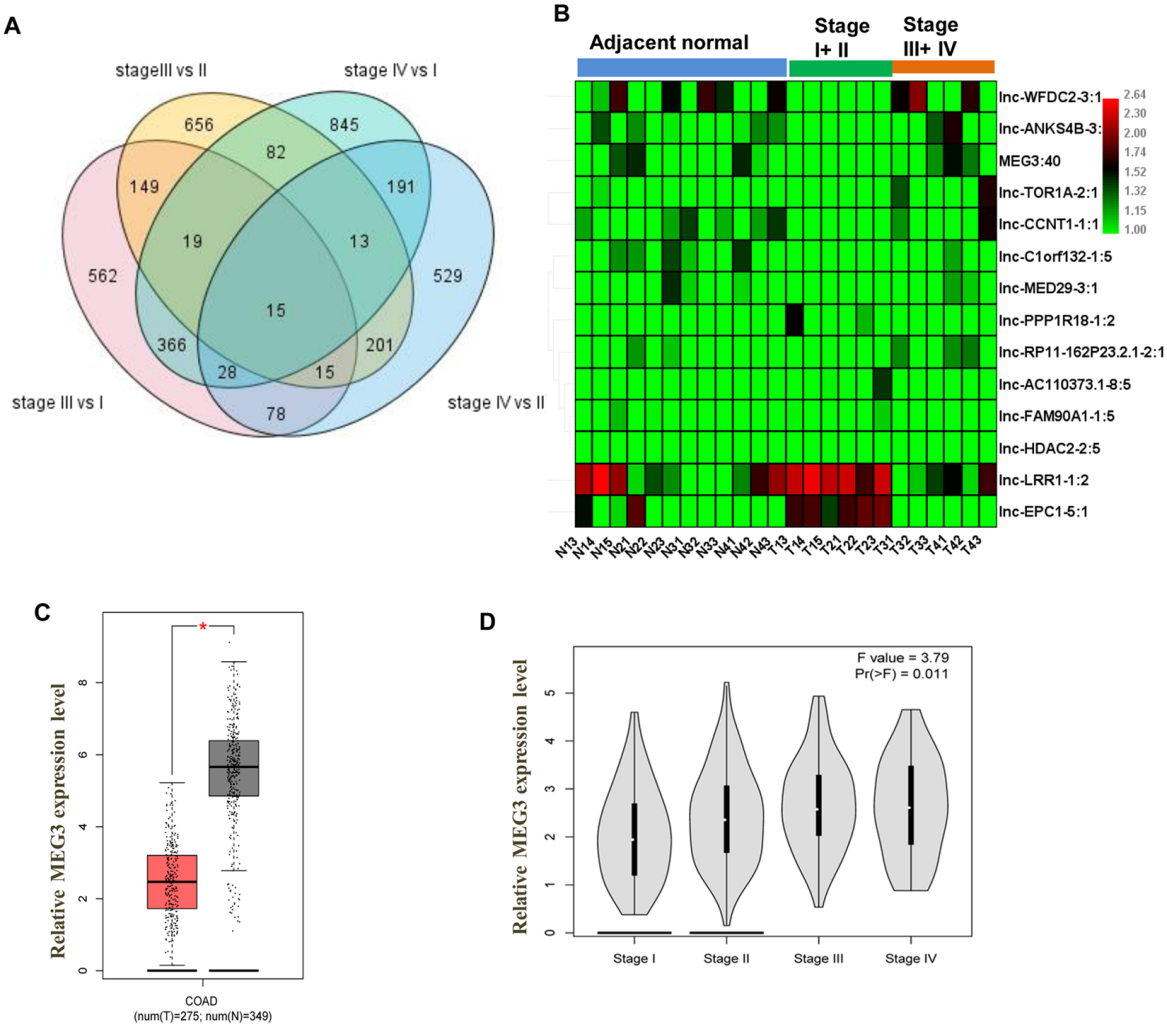
Differentially expressed (DE) lncRNAs between the stage III/IV tumor tissue and the stage I/II tumor tissue. (**A**) Venn diagram analysis of DE lncRNAs at stage III vs stage I, stage III vs stage II, stage IV vs stage I, and stage IV vs stage II of CC **(B**) Heatmaps of DElncRNAs in stage III/IV tumor. The red and green colors represent up- and down-regulation, respectively. (**C**) Expression of lncRNA MEG3 in tumor tissues of colon adenocarcinoma (COAD) and normal tissues. (**D**) Expression of lncRNA MEG3 in COAD of different stages.

We determined the DElncRNAs between the stage III/IV tumor tissue and the stage I/II tumor tissue. A total of 3749 DElncRNAs were related to the advanced stages of cancer development (stages III and IV) (Fig. 2A). Fourteen DElncRNAs were aberrantly expressed in these four groups, of which, 5 DElncRNAs were downregulated while 9 DElncRNAs were up-regulated (Fig. 2B). The expression levels of maternally expressed gene 3 (MEG3), a known lncRNA, were markedly downregulated in colon adenocarcinoma (COAD) as observed in the TCGA database (P < 0.05, Fig. 2C). More importantly, the MEG3 was differentially expressed in the stages I to IV (P < 0.05, Fig. 2D).

### Identification of DEmRNAs and DEmiRNAs

A total of 13,278 DEmRNAs were enriched in the advanced stages of cancer (stages III and IV). Sixty three DEmRNAs were aberrantly expressed in these four groups (Supplement 1A), among which, 23 DEmRNAs were downregulated while 40 DEmRNAs were up-regulated (Supplement 1B). GO functional enrichment analyses were performed to predict the functional role of the DEmRNAs among stages III and IV (Supplement 1C). We found that most DEmRNAs were enriched in the functions related to the *wound healing, cell adhesion, angiogenesis, cell proliferation, and cell differentiation* that play an important role in the progression of the cancer. Additionally, a total of 117 DEmiRNAs were identified between the stages III and IV and stages I and II. However, there was no crossover of DEmiRNAs between these four groups; the up- and down-regulated DEmiRNAs in each group are shown on the volcano map (Supplement 1D).

### WGCNA analysis of DEmRNAs and functional annotation of the modules

**Figure 3:**
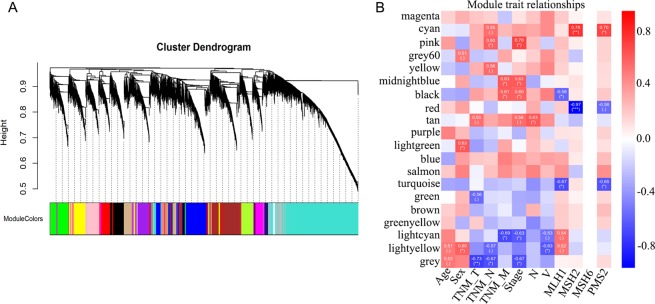
WGCNA analysis of DEmRNAs. (**A**) Cluster dendrogram and color representation of the co-expression network modules produced by average linkage hierarchical clustering of genes based on topological overlaps in the DEmRNAs. (**B**) Pearson correlation algorithm analysis of the correlation coefficient and P-value of module feature genes, clinical pathology, and demographics traits.

**Figure 4:**
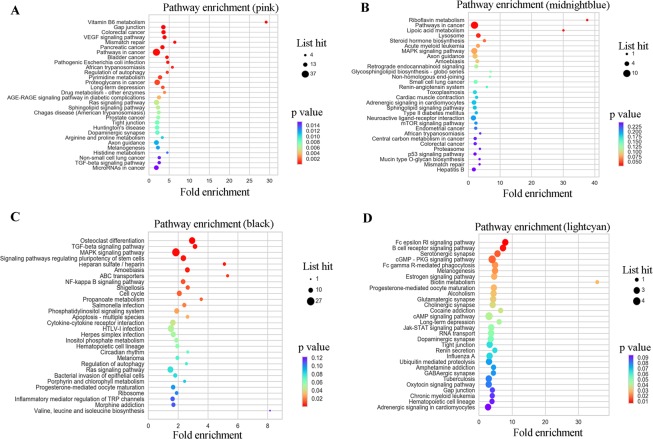
KEGG pathway enrichment analysis of core genes in different modules. (**A**) KEGG pathway enrichment analysis of core genes in pink module. (**B**) KEGG pathway enrichment analysis of core genes in midnight blue module. (**C**) KEGG pathway enrichment analysis of core genes in black module. **(D**) KEGG pathway enrichment analysis of core genes in light cyan module. The size of the ball represents the number of genes enriched in each term. The color of the ball represents the number of the P value.

To illustrate the functional enrichment of DEmRNAs in CC development, gene co-expression network of 17105 mRNAs among 12 tumor samples was constructed through WGCNA analysis. All the DEmRNAs were divided into twenty color modules (Fig. 3A); among these the grey module was designated as the gene set that cannot be attributed to any module and has no reference significance. Then, Pearson correlation algorithm was used to calculate the correlation coefficient and P-value of module feature genes and clinical pathology as well as demographics traits such as age, sex, and TNM stage. Modules related to each trait were screened with the threshold that the absolute value of correlation coefficient must be ≥0.5 and p-value should be <0.05. The results showed that the genes in the module were highly correlated with both the traits and characteristics (Fig. 3B). We surprisingly found that the mRNA expression was correlated with TNM stage as well as pathological staging in module pink, midnight blue, black, and light cyan. Module core gene analysis of pink, midnight blue, black, and light cyan is showed in Supplement 2; this figure shows the analysis of the top 50 genes with the highest degree of connectivity in each module and the relationship between these genes. Subsequently the KEGG pathway enrichment analysis was carried out to illustrate the functional and pathway enrichment of mRNAs in 4 modules. Among these four modules, most mRNAs were found to have been enriched in *VEGF signaling pathway, pathway in cancer, TGF-beta signaling pathway, MicroRNAs in cancer, MAPK signaling pathway, mTOR signaling pathway, and colorectal cancer pathway*; all these pathways are closely related to the occurrence and development of tumor (Fig. 4A–D).

### Construction of the DElncRNAs-DEmiRNAs-DEmRNAs network in CC

**Figure 5:**
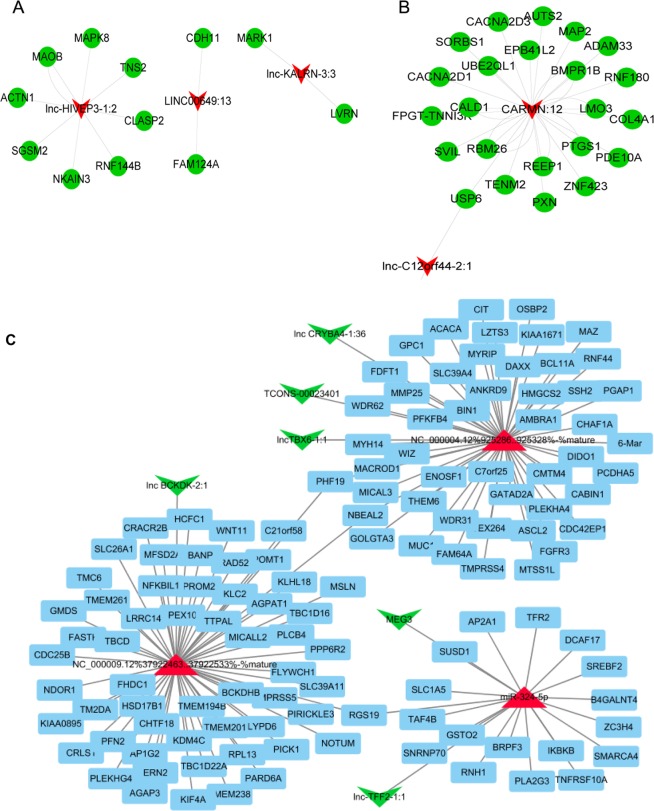
The DElncRNAs-DEmiRNAs-DEmRNAs ceRNA network. The lncRNA–mRNA network of pink (**A**) and black (**B**) module. Green balls represent DEmRNAs and red polygons represent DElncRNAs. (**C**) The DElncRNAs-DEmiRNAs-DEmRNAs ceRNA network. Red triangles represent DEmRNAs, green polygons represent DElncRNAs, and blue polygons represent DEmRNAs.

In addition to the DEmRNA co-expression analysis, Pearson correlation test was also used to determine the co-expression network of DElncRNA and DEmRNA; the candidate DElncRNA–DEmRNA network of pink and black module is presented in Fig. 5A,B, respectively. Moreover, based on the transcriptome and small RNA sequencing results, the ceRNA network was constructed with 3 DEmiRNAs, 5 DElncRNAs, and 130 DEmRNAs (Fig. 5C); the P-Value was limited to no more than 0.001. We observed that lncTBX6-1:1, lnc CRYBA4-1:36, and TCONS-00023401 might act as ceRNAs by sponging NC_000004.12%925286.0.925328%-%mature and targeting 52 mRNAs including that for Fibroblast Growth Factor Receptor 3 (FGFR3). While lnc-TFF2-1:1 and MEG3 might target 19 mRNAs such as Nuclear Factor Kappa B Kinase Subunit Beta (IKBKB) by sponging hsa-miR-324-5p, lnc BCKDK-2:1 might target 32 mRNAs such as Wnt Family Member 11 (WNT11) and Phospholipase C Beta 4 (PLCB4), by sponging NC_000009.12%37922463.0.37922533%-%mature. Moreover, several mRNAs in the ceRNA network were enriched in *pathways involved in cancer* such as WNT11, FGFR3, PLCB4, and IKBKB. Importantly, the TCGA database revealed that the expression of lncRNA MEG3 was positively correlated with Wnt11, PLCB4, and IKBKB, and negatively correlated with FGFR3 (Supplement 3).

### Verification of key lncRNAs, miRNAs, and mRNAs in CC

**Figure 6:**
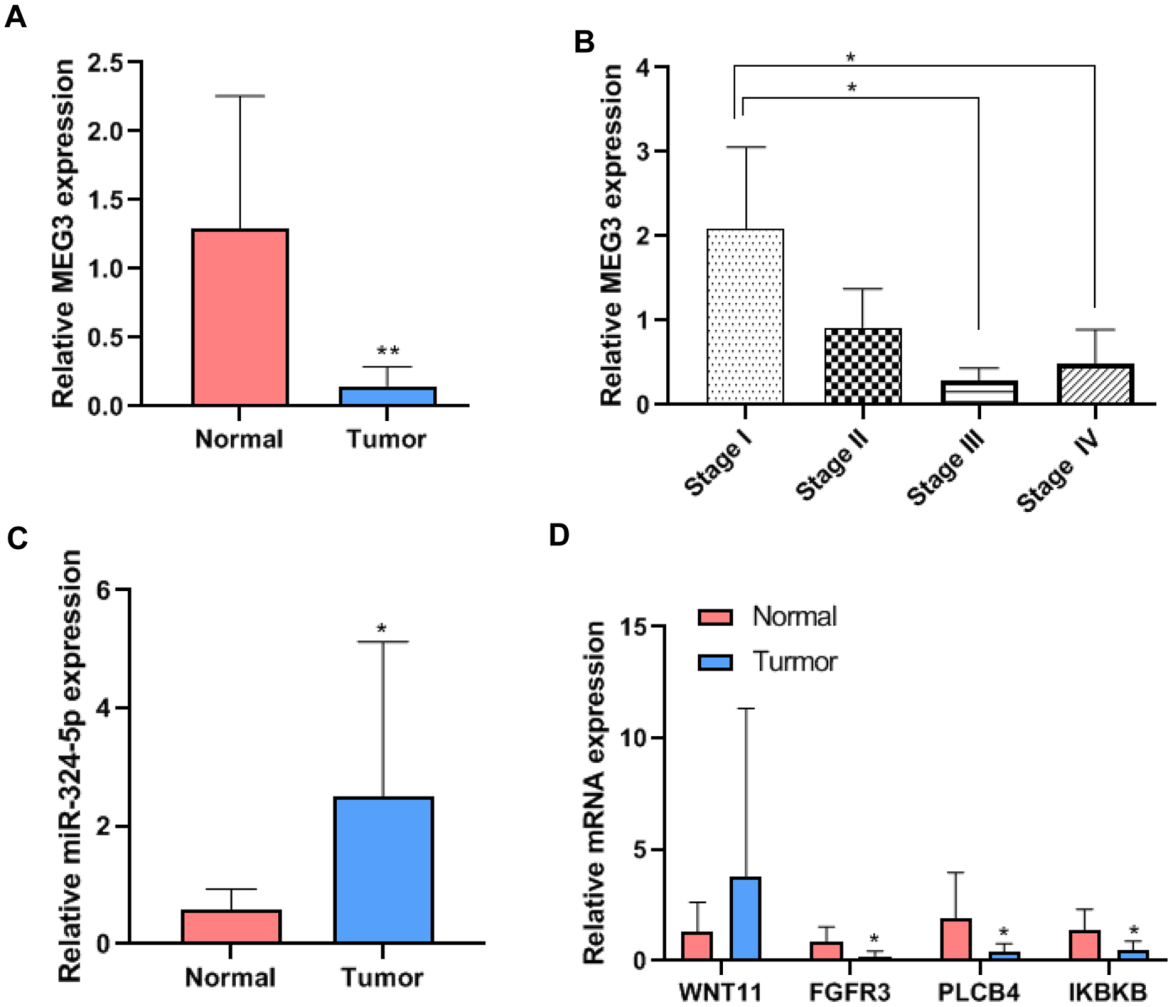
Verification of the key lncRNAs by real time PCR. (**A**) Expression of lncRNA MEG3 in tumor tissues and adjacent normal tissues. N = 40. Pair t test, **P < 0.01. (**B**) Expression of MEG3 in tumor tissues belonging to four stages. N = 10, two-tailed t test, *P < 0.05. (**C**) Expression of hsa-miR-324-5p in tumor tissues and adjacent normal tissues. N = 40. Pair t test, *P < 0.05. (**D**) Expression of mRNAs in tumor tissues and adjacent normal tissues. N = 40. Pair t test, *P < 0.05.

MEG3 was selected as a candidate key lncRNA due to its differential expression between the stages as was evident from the TCGA database and our RNA-seq. Real time PCR showed that the expression of MEG3 was downregulated in the tumor tissues as compared to the adjacent normal tissues (Fig. 6A). Moreover, MEG3 levels were decreased in tumor tissues belonging to stages III and IV as compared to those of stage I (Fig. 6B). The expression of hsa-miR-324-5p, which predictively targeted MEG3, was upregulated in the tumor tissues as compared with the adjacent normal tissues (Fig. 6C). The expression of FGFR3, PLCB4, and IKBKB were downregulated in the tumor tissues as compared to the adjacent normal tissues (Fig. 6D). These results were consistent with the results obtained from the RNA-seq. The expression of hsa-miR-324-5p and mRNAs showed no significant difference between different stages.

## Discussion

Long non-coding RNAs (lncRNAs) have been reported to act as competing endogenous RNAs (ceRNAs) and play significant roles in the development of various diseases and tumors. Several recent studies have mapped the ceRNA network of lncRNA for colon adenocarcinoma from different points of view^14,15^. Our study is first to reveal that the DElncRNAs are related to cancer staging and its ceRNA network. We also have provided several candidate DElncRNAs and related DEmiRNAs and DEmRNAs involved in different pathological stages and development of CC. These factors might act as diagnostic markers of CC staging and therapeutic targets for CC treatment.

Tumor staging is a process of assessing the number and locations of malignant tumors in the body. Tumor staging describes the severity and extent of malignancy based on the primary tumor in the individual and the degree of dissemination. Correct tumor staging helps to rationalize treatment plans, accurately determine prognosis, and evaluate efficacy; Stage I and stage II are curable while stage III and stage IV require long-term, comprehensive treatment. Our study combined with TCGA database has revealed that MEG3 is associated with tumor staging. MEG3 is downregulated and acts as a tumor suppressor in various cancer types including breast cancer, liver cancer, glioma cancer, colorectal cancer, cervical cancer, gastric cancer, lung cancer, ovarian cancer, and osteosarcoma^16,17^. For instance, MEG3 has been shown to inhibit cells proliferation and migration in CC by decreasing Clusterin expression via directly binding to Clusterin protein^18^. In gastric cancer, MEG3 has been shown to inhibit cell proliferation and metastasis via p53 signaling pathway^19^. In cholangiocarcinoma, MEG3 level was found to have been significantly decreased in cholangiocarcinoma tissues and cells as compared to that in nontumor controls and it suppressed cell proliferation and invasion via regulating Bmi1/RNF^20^. In cervical cancer, MEG3 was downregulated in the cancer tissues as compared to that in corresponding normal tissues and downregulated MEG3 was associated with recurrence and short overall survival^21^. In breast cancer, MEG3 is known to be downregulated in cancer tissues and cells and MEG3 overexpression attenuates the tumorigenesis via PI3K/Akt pathway by sponging miR-21^22^. In addition to cancer cells, MEG3 is also expressed in other cells such as immune cells and fibroblasts, and affects their gene expression patterns. MEG3 expression is upregulated in CD4+ T cells of patients with immune thrombocytopenic purpura and this enhances the immune imbalance of Treg/Th17^23^. MEG3 is downregulated in Tenon’s capsule fibroblasts as compared with control fibroblasts and is known to inhibit cell proliferation ability by interacting with Nrf2 protein^24^. Our study, for the first time reveals that the MEG3 expression is downregulated in CC and higher tumor grade is related to higher MEG3 expression, indicating that MEG3 might act as a diagnostic marker for the pathological staging.

In addition to lncRNAs, hsa-miR-324-5p and several mRNAs including WNT11, FGFR3, PLCB4, and IKBKB were revealed to be related with the pathological stage and development of CC. Hsa-miR-324-5p is associated with enhanced oncogenicity and up-expression of hsa-miR-324-5p in the colorectal cancer cells suppresses CRC tumorigenicity *in vitro* as well as *in vivo*^25^. It might act as a therapeutic target for CC treatment. Additionally, the results showed that MEG3 was positively correlated with Wnt11, PLCB4, IKBKB and negatively correlated with FGFR3 (Supplement 3). These observations provide clues for our further research based on the hypothesis that MEG3 might be involved in CC staging by regulating the expression of these genes.

Wnt protein family is known to play fundamental roles in the cancer development^26^. It was reported that Wnt-11 promoted cancer cell migration and invasion independently of β-catenin^27^. Wnt-11 is related to the degree of malignancy and metastasis of cervical cancer^28^, which is consistent with our study revealing that the Wnt-11 expression is also related to the degree of malignancy in CC. However, our study has several limitations. First, the down-regulation of MEG3 needs to be verified in the CC cell lines and the function of MEG3 should be confirmed by cell function assays. Secondly, the relationship between candidate DEmRNAs genes and MEG3 in the colon carcinogenesis will need to be confirmed by interaction validation assay.

## Materials and Methods

### Patients and samples

This study was approved by the Human Ethics Committee of First affiliated hospital of kunming medical university. Written informed consent was taken from all the patients. All methods were performed in accordance with the relevant guidelines and regulations.

A total of 40 patients with CC (with 10 patients each in stages I, II, III and IV) without anti-cancer treatment at pre-operation period were taken into the account for our study. All the cases were diagnosed pathologically and treated in the Department of Surgical Oncology, First Affiliated Hospital of Kunming Medical University in 2018. The CC samples were divided into four groups viz. stage I, II, III or IV according to the size of the tumor, infiltration range, and metastasis. Stage I and stage II are early and mild stages of cancer that are curable whereas stage III and stage IV are advanced stages of cancer that require long-term, comprehensive treatment. Grouping of samples and clinical and pathological characteristics are given in Table 1. This study was approved by the Human Ethics Committee of First affiliated hospital of kunming medical university Ethics committee. Written informed consent was taken from all the patients.

**Table 1:** Grouping and clinical characteristics of colon cancer and its adjacent samples in the RNA-seq.

Samples	Age	Sex	TNM	Stage	N	V	MLH1	MSH2	MSH6	PMS2
N13,T13	80	M	T2N0M0	I	+	−	+	+	+	+
N14,T14	62	M	T1N0M0	I	−	−	+	+	+	+
N15,T15	67	M	T2N0M0	I	+	−	+	+	+	+
N21,T21	67	F	T4aN0M0	IIB	−	−	+	+	+	+
N22,T22	68	M	T3N0M0	IIB	+	−	+	−	+	−
N23,T23	46	F	T3N0M0	IIA	−	−	−	+	+	−
N31,T31	73	F	T4aN2M0	IIIB	+	−	+	+	+	+
N32,T32	68	M	T4aN1M0	IIIB	+	+	+	+	+	+
N33,T33	54	F	T4bN2bM0	IIIC	+	+	+	+	+	+
N41,T41	41	M	T4aN1cM1b	IVB	+	+	+	+	+	+
N42,T42	50	F	T4aN1cM1b	IVB	+	+	−	+	+	+
N43,T43	65	M	T2N0M1	IVA	−	−	+	+	+	+

### Whole transcriptome sequencing

First, the total RNA of the 12 pairs of samples with 3 pairs in each stage was extracted, the ribosomal RNA was digested using the TruSeq Stranded Total RNA with ribo-zero Gold reagent, and disrupted into short fragments. Then cDNA strands were synthesized using fragmented RNA. An A tail was added to the purified cDNA and then it was connected to the sequencing connector, followed by fragment size selection, and finally PCR amplification. After the construction of RNA library, Agilent 2100 Bioanalyzer, Illumina (Illumina, CA, USA) sequencers were used for the sequencing. Total transcriptome sequencing was used not only to analyze the protein-coding genes (mRNA) but also long non-coding RNA (lncRNA).

### Identification and differential expression level analysis of lncRNAs

Stringtie (version 1.3.3b.Linux_x86_64) software^29^ was used to reconstruct the transcripts of each sample based on a probabilistic model. According to the characteristics of lncRNAs, a rigorous four-step screening method was adopted to obtain candidate lncRNAs. First, the splicing transcript was compared with the reference transcript, and the transcripts with known coding transcripts or loci were eliminated. Next, the transcripts obtained from the first step were screened according to the length greater than 200 bp and the number of exons greater than or equal to 2. Subsequently, gene coding ability was predicted for the screened transcripts in the second step and the transcripts with coding potential were removed using coding potential calculator (CPC, version 0.9-r2)^30^, coding-non-coding index (CNCI, version 1.0)^31^, the protein families database (Pfam, version 30.0)^32^, and improved k-mer scheme (PLEK, version 1.2)^33^. The lncRNA sequences obtained in step 3 were then compared with the known lncRNA by blastn software, and the repeated sequences were removed. The significant difference was defined as the log2 (fold change) >1 or log2 (fold change) <-1, and the p-value of <0.05. To identify the tumor related lncRNAs, the DElncRNAs between the cancer tissues and their adjacent normal tissues were identified for each stage, and then the common DElncRNAs in all the stages were obtained by taking the intersection elements. To identify the tumor development related lncRNAs, DElncRNAs between the advance stages (III/IV) and early stages (I/II) were analyzed in the tumor tissue including stage III versus stage I, stage III versus stage II, stage IV versus stage I, and stage IV versus stage II. The common DElncRNAs between stage III/IV and stage I/II were obtained by considering the intersection elements in the above comparison.

### Weighted gene co-expression network (WGCNA) analysis

To find clusters (modules) of highly correlated 17105 mRNAs from 12 samples, WGCNA analysis was carried out using WGCNA R package^34^. First, those genes with less variable expression levels were filtered out. Secondly, with the selected power value, a weighted co-expression network model was established. A total of 17105 genes were divided into 20 modules, among which, the grey module was the gene set that could not be assigned to any module and had no reference significance. The calculation process was measured by the block wise Consensus Modules of WGCNA package. Module analysis includes cluster graph analysis, GO and KEGG analysis etc. Then, Pearson correlation algorithm was used to calculate the correlation coefficient and P-value of module feature genes and characters. Modules related to each trait were screened according to the threshold of absolute value of correlation coefficient of >0.5 and p-value of <0.05. Finally, in the network constructed by WGCNA, the connectivity of most nodes (genes) is very low because the WGCNA is a scale-free network; the core genes in a module are the group of genes with the highest connectivity in the module.

### Small RNA sequencing and miRNA targets prediction

Total RNA was isolated from CC tissue and its adjacent normal tissue with Trizol reagent (Invitrogen, CA, USA). The RNA quantity and purity was determined by Nanodrop (Thermo, MA, USA) and 1% gel electrophoresis. Each purified RNA sample was used for the construction of RNA libraries using the miRNA Library Prep Kit (New England Biolabs, MA, USA) according to the Illumina’s manufacturer’s information. Subsequently, RNA sequencing was performed on Hiseq2500 (Illumina, CA, USA) with 50 million reads depth-datasets and the raw data were filtered with FastQC to get rid of the low-quality reads. Then high-quality reads were matched with miRBase (http://www.mirbase.org/) database to recognize the known miRNAs. The miRNAs targets were determined by Miranda and RNAhybird. The criteria for screening were score ≥150 and energy <-20(Miranda) or energy <-25(RNAhybird), and miRNA targets were obtained by taking the intersection of the Miranda and RNAhybird results.

### Gene ontology functional enrichment and Kyoto encyclopedia of genes and genomes (KEGG) pathway enrichment

Hierarchical clustering was performed using TBtools V0.655 (Toolbox for biologists). DEmRNAs were supplied to GO analysis and KEGG pathway analysis by DAVID (http://david.ncifcrf.gov). GO analysis was used to evaluated the functions of the screened DEmRNAs including biological process (BP), Molecular Function (MF), and cellular component^1^ analysis. KEGG analysis was used to map DEmRNAs to KEGG pathways. The P value of <0.05 represents significant difference.

### Construction of the DElncRNA-DEmiRNA-DEmRNA network

The lncRNAs, miRNAs, and mRNAs that were differentially expressed between the colorectal and their adjacent normal tissues were selected to establish the ceRNA network. The regulated pairs DElncRNA-DEmiRNAs and DEmiRNAs-DEmRNA were predicted by RNAhybrid and Miranda, respectively. Threshold energy of <–20 and score of >150 was used for Miranda whereas energy of <–25 was used for RNAhybrid. The common pairs of the Miranda and RNAhybrid results were selected and only the pairs displaying negatively regulated correlation were used for ceRNA network. Finally, the DElncRNA-DEmiRNA-DEmRNA network was constructed by using the Cytoscape software (version 3.6.1) and DEmiRNA was located in the transportation hub of the network.

### Gene expression profiling interactive analysis (GEPIA) of known DElncRNAs and DEmRNAs

Gene Expression Profiling Interactive Analysis (GEPIA, http://gepia.cancer-pku.cn/) was used to compare the expression of DEmRNAs or DElncRNAs between the CC tissues and the normal tissues. Threshold was set to |Log2FC| >1 and P-value < 0.01. The correlation analysis was performed by GEPIA using Pearson’s Correlation Coefficient.

### Real time PCR

The expression levels of key lncRNA, miRNA, and mRNAs were verified via real time PCR in tumor tissues and adjacent normal tissues from 40 patients (with 10 patients in each stage). The characteristics of the patients involved in the real time PCR analysis are shown in Supplement 4. Total RNA was isolated with TRIzol reagent (Invitrogen, Carlsbad, CA, USA). RNA was reverse transcribed to cDNA using a RevertAid™ First Strand DNA Synthesis Kit (Thermo Fisher Scientific, Waltham, MA, USA). For miRNAs, the cDNA was synthesized using a specific gene primer. Real time PCR was carried out by a SYBR-Green PCR kit (Roche Diagnostics, Indianapolis, IN, USA) and using an ABI Q6 (Applied Biosystems, Foster City, USA). The PCR protocol was as follow: Degeneration at 95 °C for 10 min; 45 cycles of 95 °C for 15 sec and 60 °C for 60 sec; the fusion curve was established at 95 °C for 10 s, 60 °C for 60 s, and 95 °C for 15 sec. The primer sequences are given in Supplement 5. Data was analyzed using the 2^−ΔΔCt^ method while U6 and GAPDH were used as reference genes.

### Statistical analysis

Statistical analysis was carried out using the SPSS 21.0 software (NY, USA). All the statistical data are displayed as mean value ± standard deviation obtained from three independent experiments. Differences were determined by one-way analysis of variance. Student’s t test was used for comparison between the two groups. P-value of < 0.05 was considered as statistically significant.

### Ethics approval and consent to participate

This study was approved by the Human Ethics Committee of First affiliated hospital of kunming medical university. Written informed consent was taken from all the patients.

### Consent for publication

All the listed authors have read and approved the manuscript for the publication.

## Conclusions

Our study, by combining whole transcriptome sequencing, small RNA sequencing, and WGCNA analysis, systematically constructed the lncRNA ceRNA network and picked out several markers that are of importance in the development and staging of CC, and hence, can act as diagnostic markers.

## Supplementary information

Revised Supplement file 1-5

## Data Availability

All data can be obtained by email to the corresponding author.
