# Supplementary material for: Comprehensive Analysis of the Expression Profiles of Long Non-Coding RNAs with Associated ceRNA Network Involved in the Colon Cancer Staging and Progression
Source: Sci Rep. 2019 Nov 15;9:16910. doi: 10.1038/s41598-019-52883-2 (PMC6858342; doi:10.1038/s41598-019-52883-2)
Supplement: Supplementary file 1 — Revised Supplement file 1-5 [file 41598_2019_52883_MOESM1_ESM.pdf]

Comprehensive Analysis of the Expression Profiles of Long Non-Coding RNAs with Associated ceRNA Network Involved in the Colon Cancer Staging and Progression

Meini Wu[1#], Wenliang Li[2#], Fengchang Huang[2], Jing Sun[1], Kang ping Li[2], Jiandong Shi[1], Jingyu Yang[2], Jianfang Li[1], Yanhan Li[1], Ningzhu Hu[1], YunZhang Hu[1*]

[1]Department of vaccinology,Institute of Medical Biology, Chinese Academy of Medical Sciences and Peking Union Medical College, Kunming, 650118 China;

[2]Department of Surgical Oncology, First Affiliated Hospital of Kunming Medical University,Kunming,China;

[#]Equal contributors

*Corresponding author: Dr. Yunzhang Hu

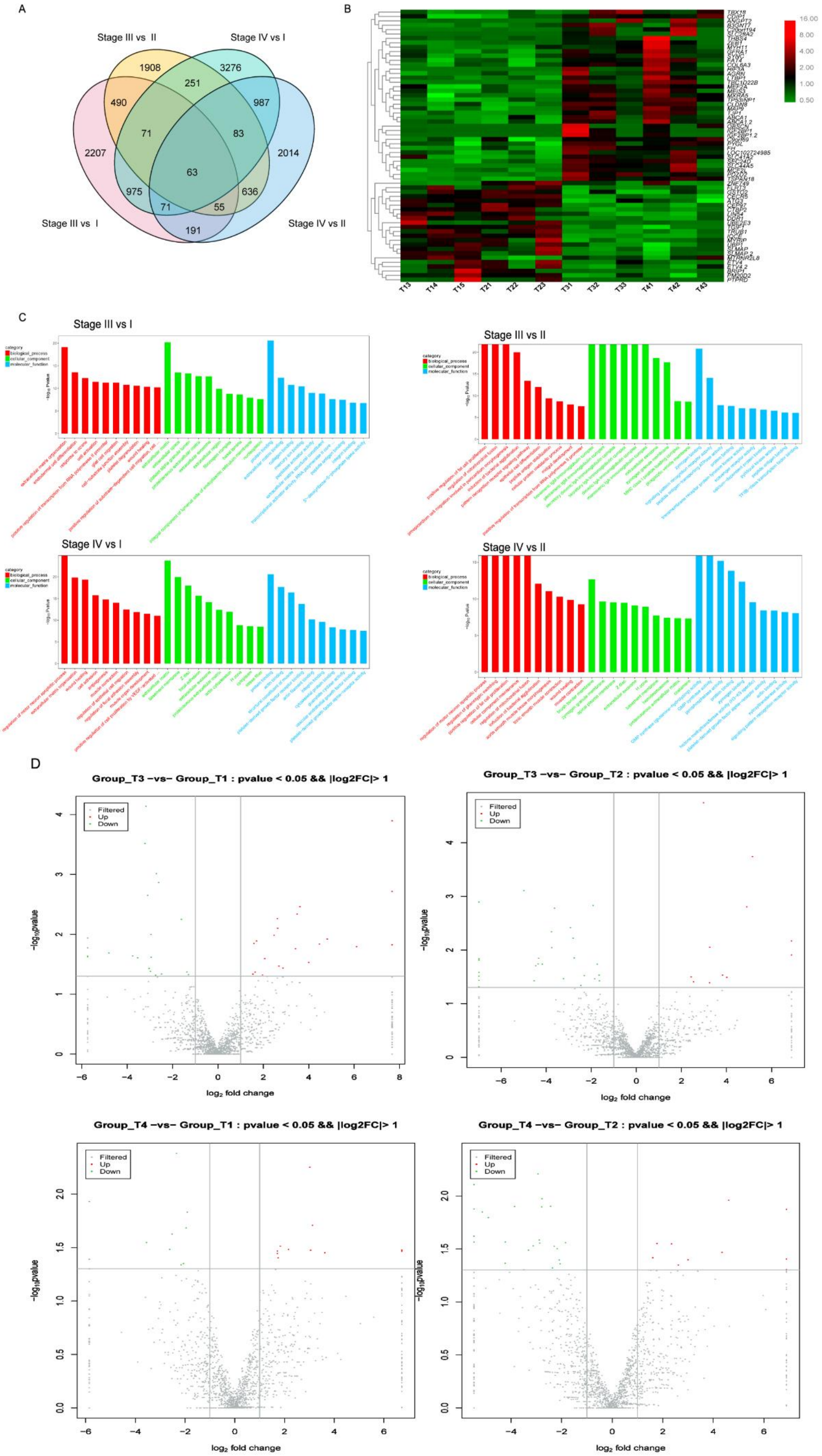
A
Stage III vs II
Stage IV vs I
Stage III vs I
Stage IV vs II
1908
3276
490
251
987
2207
71
83
2014
63
975
636
71
55
191
B
16.00
8.00
4.00
2.00
1.00
0.50
T13 T14 T15 T21 T22 T23 T31 T32 T33 T41 T42 T43
C
Stage III vs I
Stage III vs II
Stage IV vs I
Stage IV vs II
D
Group_T3 -vs- Group_T1 : pvalue < 0.05 && |log2FC|> 1
Group_T3 -vs- Group_T2 : pvalue < 0.05 && |log2FC|> 1
Group_T4 -vs- Group_T1 : pvalue < 0.05 && |log2FC|> 1
Group_T4 -vs- Group_T2 : pvalue < 0.05 && |log2FC|> 1
Filtered
Up
Down
log2 fold change
-log10pvalue

Supplement 1 Differentially expressed (DE) mRNAs and miRNAs between the stage III/IV tumor tissue and stage I/II tumor tissue. (A) Venn diagram analysis of DEmRNAs at stage III vs stage I, stage III vs stage II, stage IV vs stage I, and stage IV vs stage II of CC (B) Heatmaps of DEmRNAs in stage III/IV tumor. (C) Gene Ontology analysis of DEmRNAs. Red rectangle represents biological process (BP), green rectangle represents cellular component and blue rectangle represents molecular function (MF). (D) Volcano plot of DEmiRNAs at stage III vs stage I, stage III vs stage II, stage IV vs stage I, and stage IV vs stage II of CC.

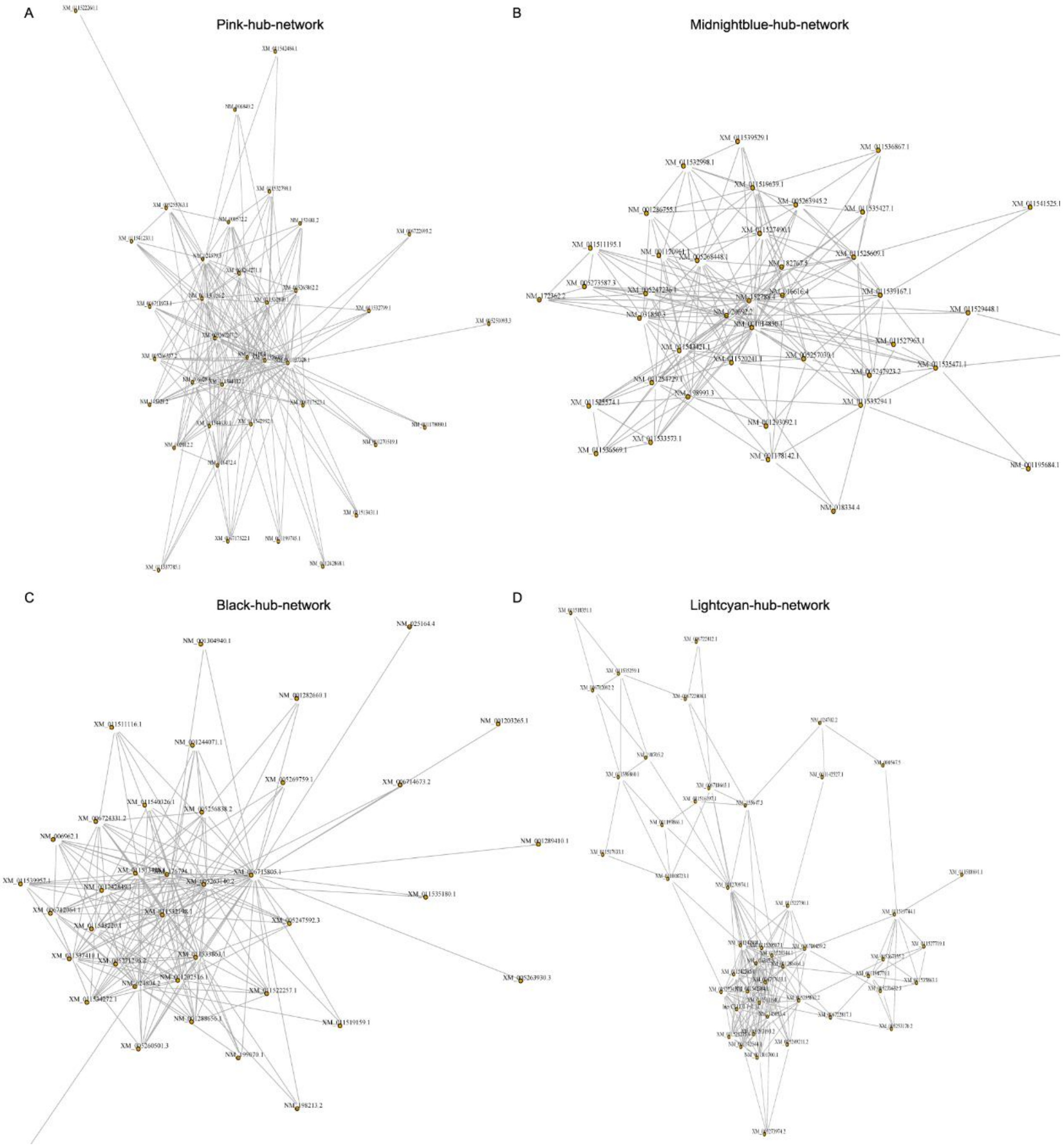

Supplement 2 Module core gene analysis of model pink, midnightblue, black and lightcyan.

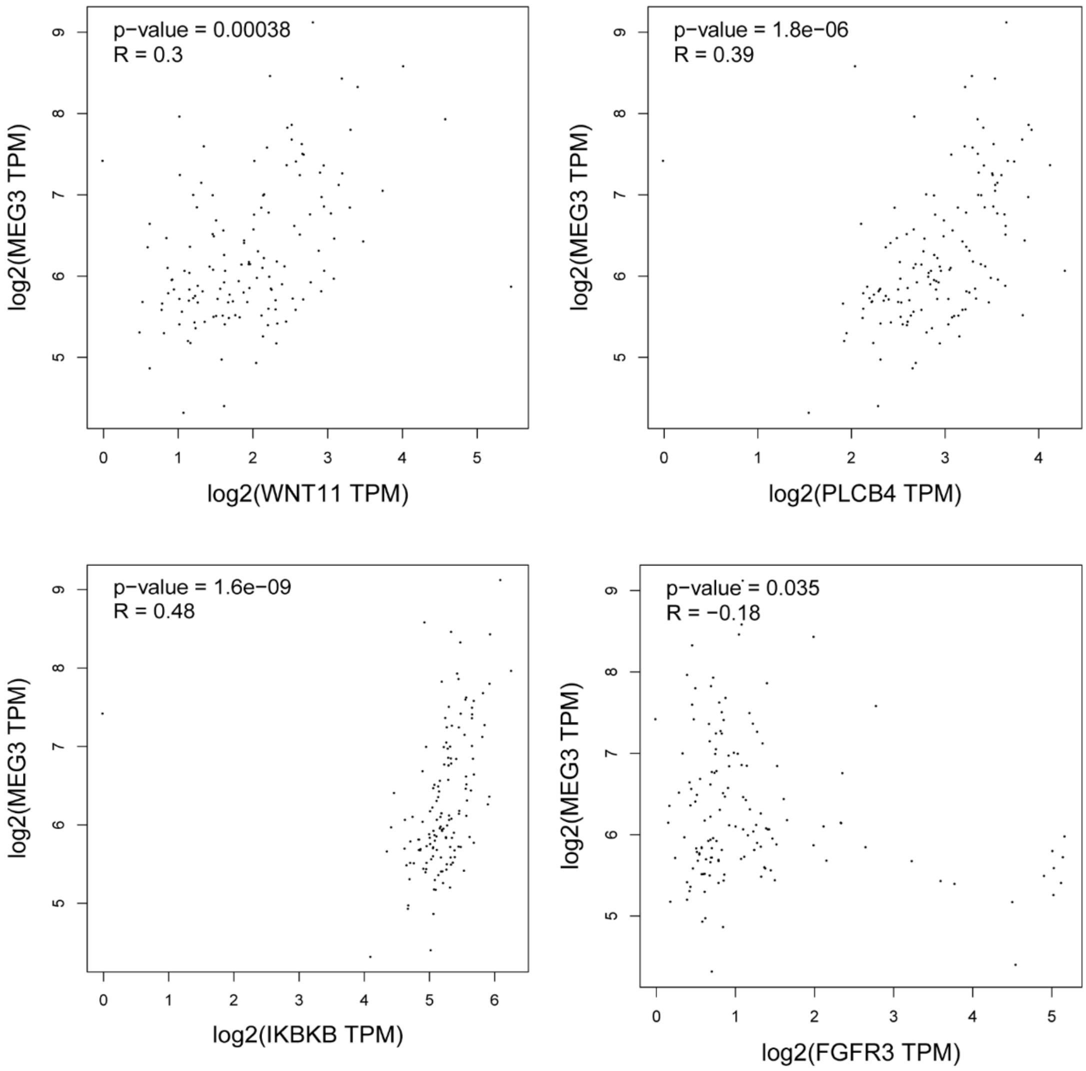

Supplement 3 The expressed relationship between lncRNA MEG3 and mRNA Wnt11, PLCB4, IKBKB and FGFR3 was analysis using the TCGA database.

Supplement 4 Demographic and clinical characteristics of colorectal cancer patients (n=40) involved in real time PCR.

Characteristics	patients， n (range)
Age	64 （41-80）
Sex	
F	24
M	16
Stage	
I	10
II	10
III	10
IV	10
T stage	
T1	3
T2	10
T3	8
T4	19
N stage	
N0	23
N1	10
N2	7
M stage	
M0	30
M1	10
N	
+	25
-	15
V	
+	12
-	28
MLH1	
+	31
-	9
MSH2	
+	39
-	1
MSH6	
+	40
-	0
PMS2	
+	35

-	5

Supplement 5 Primer sequences used in this study.

Gene name	Primer sequence
MEG3-1-F	5' GCTGAAGAACTGCGGATGGAA 3'
MEG3-1-R	CCGTGGCTGTGGAGGGATT 3'
hsa-miR-324-5p RT	5' GTCGTATCCAGTGCGTGTCGTGGAGTCGGCAATTGCACTGGATACGACCACCAAT 3'
hsa-miR-324-5p F	5' ATATACTCGCATCCCCTAGGGC 3'
hsa-miR-324-5p R	5' AGTGCGTGTCGTGGAGTCG 3'
WNT11-F	5' TTGACCTGGAGAGAGGGACC 3'
WNT11-R	5' AGGAGCATCGGAAAACTTGG 3'
FGFR3-F	5' GTGCTCAAGACGGCGGGC 3'
FGFR3-R	5' GCCACGCAGAGTGATGAGAAAA 3'
PLCB4-F	5' TGAAGGCAAGGAAGGACAGGT 3'
PLCB4-R	5' CCACTGCAGACACAAACTATCCG 3' 1
IKBKB-F	5' TGGCACAATCAGGAAACAGGT 3'
IKBKB-R	5' GGGTGGGTCAGCCTTCTCAT 3'
GAPDH -F	5' AGAAGGCTGGGGCTCATT 3'
GAPDH -R	5' TGCTAAGCAGTTGGTGGTG 3'
U6-F	5’ CGATACAGAGAAGATTAGCATGGC 3’
U6-R	5’ AACGCTTCACGAATTTGCGT 3’ 3’
